# Acute Exacerbation of Chronic Periapical Pathology in a Dentist: An Autobiographical Case Report

**DOI:** 10.7759/cureus.100715

**Published:** 2026-01-03

**Authors:** Ramya Sekar, Thangadurai Maheswaran, Mathimaraiselvan Chittrarasu, Sethuraman Vijayparthiban, Ramamurthy Suresh

**Affiliations:** 1 Oral, Maxillofacial Pathology and Oral Microbiology, Meenakshi Ammal Dental College and Hospital, Meenakshi Academy of Higher Education and Research (Deemed to be University), Chennai, IND; 2 Oral Pathology and Microbiology, Adhiparasakthi Dental College and Hospital, Melmaruvathur, IND; 3 Conservative Dentistry and Endodontics, Vivekanandha Dental College for Women, Tiruchengode, IND; 4 Oral and Maxillofacial Surgery, Thanjavur Medical College, Thanjavur, IND; 5 Orthodontics, Thanjavur Medical College, Thanjavur, IND

**Keywords:** acute dental pain, analgesic rotation, autobiographical case report, calcium hydroxide, endodontic therapy, nsaid, odontogenic infection, periapical abscess, periapical granuloma, self-medication

## Abstract

Acute exacerbations of chronic periapical pathology require prompt surgical or endodontic intervention, supported by pharmacotherapy. The role of analgesic selection, escalation, and rotation in acute dental pain remains underexplored. A 39-year-old male dentist with a 27-year history of trauma to the lower left central incisor presented with acute pain of three hours’ duration. The tooth was non-vital with a stable periapical granuloma documented for over 15 years. Self-medication began with paracetamol, escalating to ibuprofen, aceclofenac, and etoricoxib, when pain worsened. Amoxicillin and metronidazole were also taken for infection control. On day three, endodontic access without anesthesia yielded minimal pus drainage but immediate pain relief. The canal was initially left open, then medicated with calcium hydroxide, and finally obturated with gutta-percha and zinc oxide-eugenol sealer. Three years of follow-up showed no recurrence. This case demonstrates symptom-driven analgesic escalation and de-escalation in an informed patient and raises the concept of analgesic rotation for reducing cumulative toxicity. While this may be relevant in chronic pain management, its role in acute odontogenic pain is limited, especially for agents with shared adverse profiles such as non-steroidal anti-inflammatory drugs (NSAIDs). Timely endodontic intervention remains the cornerstone of treatment for acute periapical abscesses. Analgesic prescribing should be individualized and symptom-based rather than fixed-duration, with rotation considered selectively.

## Introduction

Chronic periapical periodontitis, which often manifests radiographically as periapical radiolucency, is a common sequel of pulp necrosis. It represents a localized inflammatory response of the periapical tissues to microbial invasion of the root canal system [1]. These lesions are often asymptomatic for years and are incidentally found on routine radiographs. The histopathological correlate is often a periapical granuloma, which is a zone of granulation tissue surrounded by a fibrous capsule attempting to wall off the infection [2].

The transition from a chronic, asymptomatic state to an acute, symptomatic exacerbation is a well-documented phenomenon [3,4]. This shift is typically triggered by a change in the host-microbe relationship, leading to a rapid increase in inflammatory mediators, tissue edema, and pus formation, resulting in a clinical picture of an acute apical abscess [5]. The associated pain is often severe, constant, and throbbing, exacerbated by percussion and palpation as pressure builds within the rigid confines of the alveolar bone.

The management of such acute exacerbations follows established principles: removal of the cause (pulpal debris and microbes), establishment of drainage, and infection control. While antibiotics are indicated in the presence of systemic signs (e.g., fever, lymphadenopathy) or spreading infection, the cornerstone of treatment remains local intervention, primarily through access opening and chemomechanical debridement of the root canal system [6,7]. Analgesics, particularly non-steroidal anti-inflammatory drugs (NSAIDs), play a crucial role in managing the inflammatory pain component [8].

This report presents an autobiographical account of an acute exacerbation in a dental surgeon. This unique perspective offers insights into the natural history of the disease, the decision-making process of a healthcare professional acting as his own patient, and the empirical pharmacological management undertaken before definitive treatment. Furthermore, it explores the clinical reasoning behind a self-imposed "analgesic rotation" strategy and the challenges of adhering to professional treatment protocols when experiencing severe pain.

## Case presentation

The patient was a 39-year-old male dental surgeon and postgraduate in oral pathology, in good general health, with no significant medical history or known drug allergies.

Dental history and background

The history dated back to a traumatic injury to the lower anterior region approximately 27 years prior during adolescence. The lower left central incisor [tooth #31, FDI World Dental Federation (FDI) notation] became non-vital and progressively discolored. The patient, who was then a layperson, did not seek dental consultation until he commenced undergraduate dental training in 2000. During his studies, he became aware of the non-vital status of the tooth and the associated periapical radiolucency, which he understood to represent chronic apical periodontitis or periapical granuloma. Serial radiographs taken over the years (approximately 15, 10, and five years before the acute event) consistently showed a stable, well-defined periapical radiolucency of 2-3 mm in diameter with loss of the lamina dura. The absence of symptoms and the non-progressive nature of the radiolucency were reassuring, leading to a conscious decision to adopt a "wait-and-watch" approach rather than pursuing elective endodontic therapy. The patient rationalized this decision based on his professional access to dental care and confidence that any acute exacerbation could be managed promptly.

Presentation of acute exacerbation

In September 2022, on a Saturday morning (day one), the patient experienced a mild, dull ache localized to the lower-left central incisor region. Over the next three hours, the discomfort persisted. Since it was a weekend and he was initially reluctant to take medication due to his belief in potential side effects, he attempted to endure the pain. However, by noon, the increasing pain prompted him to take 500 mg of paracetamol in the afternoon, which was chosen as a perceived "safe" first-line analgesic. The pain continued to intensify, leading him to leave his evening practice a couple of hours early. A second dose of paracetamol (650 mg) was administered at that time, with minimal effect. Clinically, based on the history and symptoms, the patient self-diagnosed an acute exacerbation of chronic periapical pathology, likely evolving into an acute apical abscess. He concluded that access opening and drainage were the most effective interventions for pain relief. He contacted his trusted endodontist, a friend and colleague with over ten years of experience. However, the endodontist was unavailable until Monday morning because of a prior commitment. This delay necessitated self-management for approximately 48 hours.

Self-management and pharmacological intervention

The pain escalated significantly by day two, becoming severe and throbbing. The patient searched his home supply and found ibuprofen 400 mg tablets, which he took after breakfast. Owing to pain, he cancelled his planned Sunday clinic. The pain remained unbearable by the afternoon, prompting the administration of a second 200 mg dose of ibuprofen after lunch.

Recognizing the limitations of over-the-counter analgesics and convinced of an active infective process, the patient visited his dental clinic in the evening to procure a more robust pharmacological arsenal. He was administered aceclofenac (100 mg), amoxicillin (500 mg), and metronidazole (400 mg) in the early evening. No new radiographs were taken because the clinical diagnosis was deemed certain. When the pain persisted unabated a few hours later, he took a 60 mg dose of etoricoxib in the late evening. This combination allowed the patient to sleep.

Upon waking on day three, the pain persisted, although with a slightly reduced intensity. He took another 60 mg dose of etoricoxib after breakfast and continued the antibiotics. This firsthand experience of severe, unremitting dental pain provided a profound and empathetic understanding of his patients' suffering, a perspective previously held only theoretically.

Definitive treatment and outcome

The patient presented to the endodontist on day three as scheduled. An emergency access opening was created in tooth #31 without the use of local anesthesia. The rationale for foregoing anesthesia was to allow for immediate feedback on pain during the procedure. The initial penetration of the file beyond the apex caused sharp, transient pain, which was immediately followed by dramatic and profound relief of chronic, severe throbbing pain. A minimal amount of pus (less than a drop) was observed. At the patient's request, the canal was left open to facilitate continued drainage. No swelling or sinus tract was observed. 

The following day, on day four, the canal was gently explored, irrigated with saline, and a closed dressing was placed using calcium hydroxide as an intracanal medicament. At this time, the pain was insignificant, and the patient self-discontinued all analgesics, believing that they were no longer required for symptomatic control. The patient continued to receive antibiotics (Amoxicillin and Metronidazole) for another two days, till day five. A summary of the medication intake is presented in Table 1.

**Table 1:** Timeline of Analgesic and Antibiotic Intake

Day	Time	Medication(s) Taken
Day 1	Afternoon	Paracetamol 500 mg
Day 1	Evening	Paracetamol 650 mg
Day 2	Morning & Afternoon	Ibuprofen 400 mg
Day 2	Evening	Aceclofenac 100 mg, Amoxicillin 500 mg, Metronidazole 400 mg
Day 2	Night	Etoricoxib 60 mg
Day 3	Morning	Etoricoxib 60 mg, Amoxicillin 500 mg, Metronidazole 400 mg
Day 3	Evening	Etoricoxib 60 mg, Amoxicillin 500 mg, Metronidazole 400 mg (Analgesics stopped)
Day 4	Morning & Evening	Amoxicillin 500 mg, Metronidazole 400 mg
Day 5	Morning & Evening	Amoxicillin 500 mg, Metronidazole 400 mg (All medications stopped)

On day seven, biomechanical preparation was completed using hand instruments and saline irrigation, employing a traditional step-down technique to conserve the tooth structure for future conservative restoration. A further closed dressing with calcium hydroxide as an intracanal medicament was then applied. The remaining sign was mild tenderness on percussion of the tooth and palpation of the periapical region.

**Figure 1:**
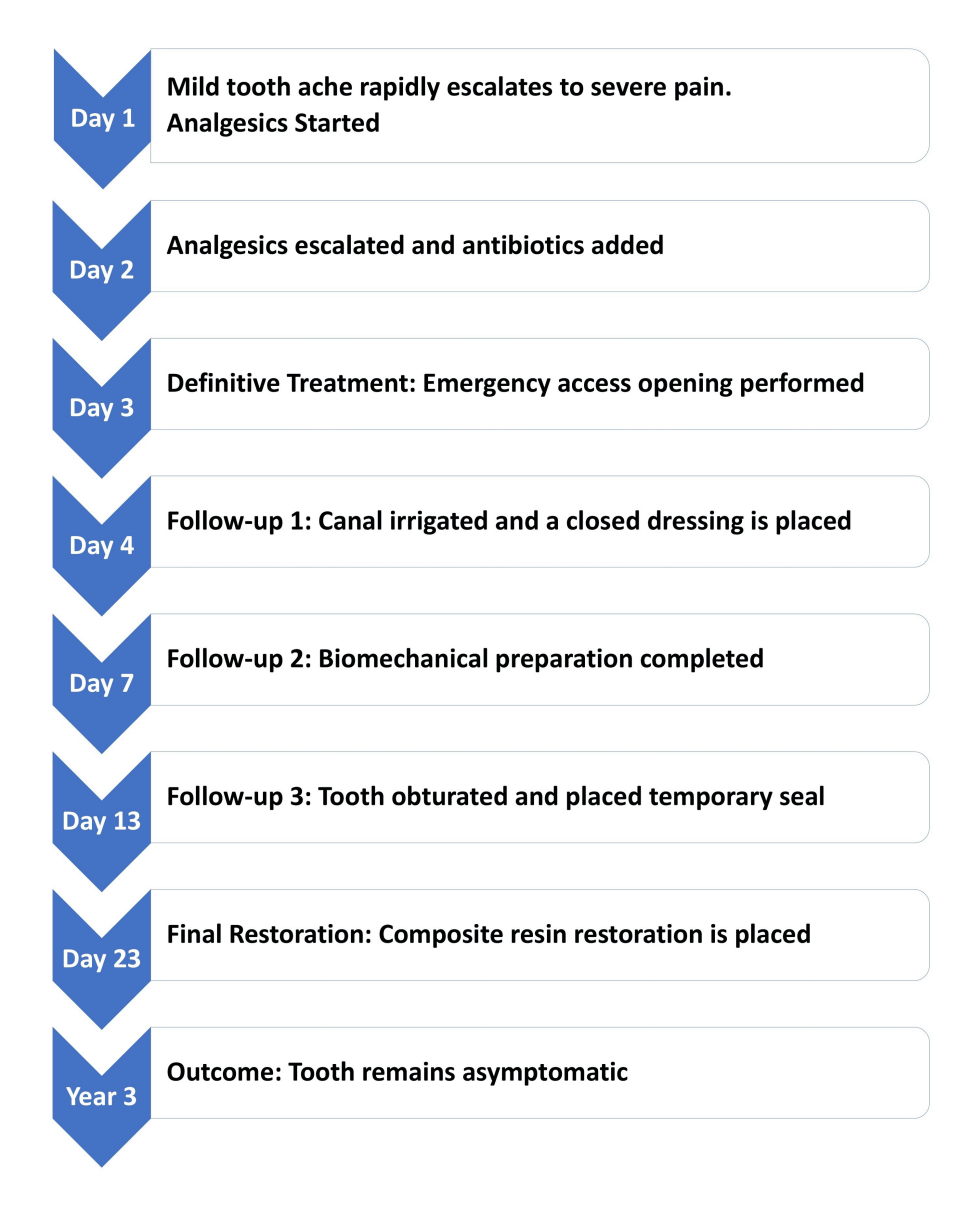
Overall Sequence of Events

On Day 13, the tooth was obturated with 2% Gutta-Percha points and a zinc oxide eugenol sealer using the cold lateral condensation technique. A temporary coronal seal was then placed. On Day 23, the tooth was restored with a definitive composite resin restoration. The patient reported no pain or discomfort at any follow-up point during the subsequent three years. The overall sequence of events is illustrated in Figure 1.

## Discussion

This autobiographical account provides a vivid narrative of the pathogenesis, progression, and management of an acute exacerbation of chronic periapical lesions. This underscores several key clinical and behavioral points relevant to dental practice.

The natural history of the presented case aligns perfectly with the established understanding. The initial trauma led to pulp necrosis, followed by the development of a chronic periapical lesion that remained in a state of equilibrium with the host's immune response for over two decades. The shift to an acute abscess likely results from a disruption of this fragile balance, potentially due to an increase in microbial virulence or a transient depression in local host defenses [3,4]. The rapid escalation of pain is a classic feature driven by the buildup of purulent exudate and inflammatory mediators (e.g., prostaglandins and cytokines) in the non-compliant periapical space, leading to tissue pressure that exceeds the pain threshold [8].

A striking aspect of this case is the author's initial neglect of the non-vital tooth. Despite his professional knowledge, he exhibited a common human bias: the "optimism bias," where individuals believe they are less likely to experience negative health events than others [9]. His access to dental care likely contributed to this deferred management, a phenomenon sometimes observed in healthcare professionals who self-triage [10]. This case serves as a reminder that knowledge does not always translate into action, especially when the patient is concerned.

The self-managed pharmacological regimen offers a real-world example of escalating pain management. The initial use of paracetamol, a centrally acting analgesic with weak anti-inflammatory properties, was suboptimal for managing a primarily inflammatory pain state [8]. The subsequent switch to ibuprofen, an NSAID, was a more pathophysiologically sound decision, as NSAIDs are highly effective for dental pain because they inhibited cyclooxygenase (COX) and subsequent reduction in prostaglandin synthesis [11]. The final escalation to more potent NSAIDs (Aceclofenac, Etoricoxib, a COX-2 selective inhibitor) reflects the clinical reality of titrating analgesic therapy to pain severity [12].

This experience raises the question posed by the author: Is there a role for structured "analgesic rotation" in acute dental pain? In chronic pain management (e.g., cancer pain), opioid rotation is practiced to improve efficacy and reduce side effects [13]. However, for acute inflammatory dental pain, the evidence base for rotating different NSAIDs or between NSAIDs and Paracetamol is limited. Clinical progression usually dictates a step-up or step-down approach based on potency and formulation, not necessarily a rotation between different drug classes to mitigate subclinical adverse effects [14]. In this case, rotation was driven by availability and escalating pain intensity rather than a planned strategy. While rotating drugs with dissimilar adverse effect profiles could theoretically minimize the risk of any single toxicity, most NSAIDs share common side effects, such as gastrointestinal irritation [15]. Therefore, a fixed-dose combination or a single potent NSAID prescribed for a short duration, as per guidelines, remains the standard of care [16,17]. Although the author's ad hoc regimen was effective, it is not recommended for patient guidance without professional consultation.

The decision to use antibiotics (Amoxicillin and Metronidazole) was based on the clinical diagnosis of an acute abscess. While current guidelines strongly emphasize that the primary treatment for a localized acute apical abscess is local drainage and debridement, with antibiotics reserved for systemic involvement [6,7], the author's decision reflects a common clinical behavior, even among professionals, when faced with severe symptoms. The prompt discontinuation of all medications once symptoms resolved, against the endodontist's advice, also mirrors a well-documented patient behavior of non-adherence to full antibiotic courses once feeling better, a practice whose implications are debated in modern medicine [18].

The most definitive moment in management was the opening of the access without anesthesia, which provided instantaneous relief. This validates the core endodontic principle that drainage and decompression of the peri-radicular tissues are the most effective means of relieving the pain of an acute apical abscess. The use of calcium hydroxide intracanal medicament between appointments is supported by the literature for its broad-spectrum antimicrobial activity and ability to suppress periapical inflammation, promoting healing [19].

Finally, the successful long-term outcome with traditional hand instrumentation and a conservative access cavity highlights that excellent results can be achieved with a meticulous technique, prioritizing the preservation of the tooth structure, which is a cornerstone of modern minimally invasive dentistry.

Limitations

This report describes a single autobiographical case and is inherently limited by its anecdotal nature and the lack of generalizability. The absence of contemporaneous diagnostic imaging and objective pain scoring restricts the ability to correlate symptom severity with disease progression or treatment responses. Pharmacological decisions were influenced by self-management, availability of medications, and individual perception of pain rather than standardized protocols, limiting the extrapolation to routine clinical practice. The concept of analgesic rotation discussed herein is observational and not supported by controlled evidence for acute odontogenic pain. Accordingly, the findings should be interpreted as reflective insights rather than as prescriptive clinical guidance.

## Conclusions

This autobiographical case report provides a unique first-person perspective on the journey from asymptomatic chronic periapical pathosis to acute debilitating pain and subsequent resolution through definitive endodontic therapy. This illustrates several key lessons.

Firstly, the "wait-and-watch" approach for known periapical pathology, while sometimes valid, carries an inherent risk of acute exacerbation, even after decades of stability. Healthcare professionals are not immune to cognitive biases and may delay their own necessary treatment, despite possessing the requisite knowledge. Secondly, the management of escalating pain often leads to empirical multi-analgesic use before definitive care. While the concept of "analgesic rotation" is intriguing for potentially mitigating cumulative side effects, its application in acute dental pain requires more robust clinical evidence. Finally, the paramount importance of local intervention (access opening and drainage) for immediate pain relief in acute apical abscesses cannot be overstated, reaffirming the established endodontic principles.

This personal experience has profoundly deepened the author's empathy for patients suffering from dental pain and serves as a compelling narrative of the clinical and human dimensions of oral disease.
